# Quality and availability of consumer information on heart failure in Australia

**DOI:** 10.1186/1472-6963-8-255

**Published:** 2008-12-12

**Authors:** Agnes I Vitry, Susan M Phillips, Susan J Semple

**Affiliations:** 1 Quality Use of Medicines and Pharmacy Research Centre, Sansom Institute, University of South Australia, Adelaide, Australia; 2 National Health and Medical Research Council's National Institute of Clinical Studies, Melbourne, Australia

## Abstract

**Background:**

Provision of consumer information and patient education are considered an essential part of chronic disease management programmes developed for patients with heart failure. This study aimed to review the quality and availability of consumer information materials for people with heart failure in Australia.

**Methods:**

The availability of consumer information was assessed through a questionnaire-based survey of the major organisations in Australia known, or thought, to be producing or using consumer materials on heart failure, including hospitals. The questionnaire was designed to explore issues around the use, production and dissemination of consumer materials. Only groups that had produced consumer information on heart failure were asked to complete the totality of the questionnaire.

The quality of information booklets was assessed by using a standardised checklist.

**Results:**

Of 101 organisations which were sent a questionnaire, 33 had produced 61 consumer resources on heart failure including 21 information booklets, 3 videos, 5 reminder fridge magnets, 7 websites, 15 self-management diaries and 10 self-management plans. Questionnaires were completed for 40 separate information resources. Most had been produced by hospitals or health services. Two information booklets had been translated into other languages. There were major gaps in the availability of these resources as more than half of the resources were developed in 2 of the 8 Australian states and territories, New South Wales and Victoria.

Quality assessment of 19 information booklets showed that most had good presentation and language. Overall eight high quality booklets were identified. There were gaps in terms of topics covered, provision of references, quantitative information about treatment outcomes and quality and level of scientific evidence to support medical recommendations. In only one case was there evidence that consumers had been involved in the production of the booklets.

**Conclusion:**

Key findings arising from the study included the need to develop a nationally coordinated approach for increasing the dissemination of information resources on heart failure. While the more recent publication of a booklet by the National Heart Foundation may have improved the situation, dissemination of written information materials may remain sub-optimal, especially among patients who are not enrolled in chronic heart failure management programmes.

## Background

Heart failure is a chronic and complex condition with high morbidity and mortality. It represents a major reason for seeking health care, accounts for a high proportion of health care expenditure, and is expected to continue to rise in prevalence, especially among older people [1]. Hospitalisations for heart failure may be caused by sub-optimal medication management or inadequate discharge planning but also by the patients' failure to adhere to medical recommendations and to engage in self-care behaviour in order to prevent further deterioration [2]. Poor patient knowledge is associated with poor prognosis in patients in heart failure [3]. The implementation of self-management programmes including patient education has been shown to be successful in improving health outcomes. A recent systematic review of randomised controlled trials of self-management interventions showed that self-management decreased all-cause and heart failure hospital readmissions significantly [4]. While it is difficult to assess the specific effect of patient information [2], the development of patient information resources is one of the first steps of any disease or self-management programme for patients with heart failure. Several studies have shown, however, that consumer information materials in general may present serious shortcomings [5,6]. They may omit relevant data, fail to give a balanced view of the effectiveness of different treatments, and ignore uncertainties [5]. They may have been developed without consumer input, with consequent deficiencies apparent [7].

Another critical issue is the availability and the dissemination of consumer resources. Several studies have shown that outpatients with heart failure may lack appropriate information and/or knowledge on their condition [2]. In New Zealand, a study found that, in a random sample of 62 patients receiving care for chronic heart failure in 30 different medical practices, 40% of the patients did not understand the nature and seriousness of their condition [8]. An American study showed that, out of 113 patients surveyed, approximately 40% of the patients did not recognize the importance of weighing themselves daily [9].

The main objectives of this study were to assess the quality of consumer information materials on heart failure developed in Australia and to evaluate their availability and dissemination.

## Methods

The availability and dissemination of consumer information materials was assessed through a survey of the major organisations or groups in Australia known, or thought, to be producing or using consumer materials on heart failure. Of 136 groups or organisations contacted initially, 101 groups indicated they had either produced heart failure resources, were in the process of producing resources or were using resources produced by other organisations, and were sent the questionnaire. This included 71 major public and private hospitals in each of the 8 Australian states and territories, 23 area health services, 1 private health insurer, 3 professional organisations and 3 drug companies. The questionnaire was emailed to a specific contact person within each of the identified groups where possible. Organisations that did not respond to the initial mail-out of the questionnaire were followed up with at least one email or telephone message. Requests for participation were also posted in electronic newsletters or discussion groups such as the "Network news" for National Prescribing Service (NPS) facilitators in the divisions of general practice and the special interest group of Chronic Heart Failure coordinators in Victoria. The survey was conducted from March to June 2003.

The questionnaire was designed to explore issues around the use, production and dissemination of consumer materials, including the type of material produced (brochure, videotape, audiotape, web-based resource), the level of consumer input into the production process, the source of funding, the target groups, the mechanisms used to disseminate the materials, the range of settings to which the materials were distributed, the success of dissemination practices, the organisation's willingness to share materials as an education resource, the possible barriers to the sharing of materials, and the use of heart failure resources produced by other groups. The level of consumer input was categorised as high (involvement of consumers at each stage of the production process), moderate (involvement of consumers at one stage at least) or no input. A pilot version of the questionnaire was tested by three health professionals working in the heart failure area and the final version incorporated their suggestions.

Only groups that had produced consumer information on heart failure were asked to complete the totality of the questionnaire. They had to fill in a separate questionnaire for each consumer material produced. The groups that had not produced consumer information were asked to answer whether they were currently using consumer information materials produced by other groups and whether they had any suggestions for improving the dissemination of consumer information.

The study protocol was approved by the Human Research Ethics Committee, University of South Australia. Participants who indicated that their group/organisation had produced consumer resources about heart failure were asked to provide, where possible, copies of the material developed to the study team. Twenty-six government or government-funded health websites were also searched to identify any consumer information on heart failure.

All materials received were categorised by type of resource. The quality of information booklets was assessed by using the "*Checklist for Assessing Written Consumer Health Information*", developed by the Centre for Clinical Effectiveness and the Health Issues Centre for the Department of Human Services in Victoria [7]. It consisted of 43 questions divided into 4 sections on presentation, language, content and treatment options. The overall quality of information booklets was rated from 1 (high quality with very few shortcomings) to 5 (poor quality with very serious shortcomings). A narrative review of the information booklets in terms of their overall presentation and the range of topics included was also undertaken. Data from questionnaires and quality assessments were analysed with the SPSS statistical software and descriptive statistics were produced.

## Results

### Analysis of materials produced

A total of 64 questionnaires were returned by 50 groups (50% response rate) as separate questionnaires had to be filled in for different resources produced by the same group. Fifty-one groups did not return the questionnaire. Thirty-three groups had produced 61 consumer resources on heart failure including 21 information booklets, 3 videos, 5 fridge magnets, 7 websites, 15 self-management diaries and 10 self-management plans (Table 1). Most information producers were hospitals or health services (79%), mainly as an outcome of specific State or Commonwealth grants, drug companies (9%), non-profit organizations (6%) and state Government's websites (6%). The main consumer resources in Western Australia and Victoria were partially or totally funded by drug companies as were all the videotapes identified.

**Table 1:** Type of consumer information materials produced

	**National**	NSW*	NT*	QLD*	SA*	TAS*	VIC*	ACT*	WA*	**Total**
Information booklet or leaflet	3	8		2	3		3		2	21
Videotape	1						1		1	3
Fridge magnet	2	3								5
Website	2			1			2	1	1	7
Self-management diary	1	5		2			5		2	15
Self-management action plan		4		2			3		1	10
Total	9	20	0	7	3	0	14	1	7	61

*NSW New South Wales; NT Northern Territory; QLD Queensland; SA South Australia; TAS Tasmania; VIC Victoria; ACT Australian Capital Territory; WA Western Australia

Questionnaires were completed for 40 separate information resources. Most materials (69%) had been produced in the last 30 months before the survey. Consumers had high input into the production of 7 resources (18%), moderate input in 18 (45%) and no input in 14 (35%). Two information booklets had been translated into 4 and 8 other languages respectively. No consumer resources had been developed specifically for Australian indigenous people. Most self-management diaries included a weight self-monitoring record. Some also included a medication record, a home exercise monitoring record and a medical appointment record. The 3 videotapes featured interviews with health professionals, topical presentations or experiences of people with heart failure. Other resources identified included a patient information pathway leaflet with information on consultations, investigations, medications, treatments, education and discharge planning from day one through to discharge and a one-page computer printout from a prescribing software.

The availability of consumer resources between geographical areas was found to vary. More than half of the consumer resources had been developed in 2 of the 8 Australian states and territories, New South Wales and Victoria. No consumer resources were identified in 3 states/territories, the Northern Territory, Tasmania and the Australian Capital Territory (ACT), with the exception of a limited website resource in the ACT. Resources were mostly distributed locally (37%) or regionally (26%) (Table 2). Some regional hospitals appeared to have a limited range of resources available, even in states such as New South Wales and Victoria with the most resources. By contrast, the resources produced by a collaborative group of hospital and primary care clinicians in Queensland had been distributed widely in this state, including among Divisions of General Practice. Hospital departments, outpatient clinics and rehabilitation centres were the most common settings where consumer materials were distributed (Table 2). No resource was available in community pharmacy and a limited number in general practitioner (GP) surgeries. Consumer materials were most frequently handed out directly by health professionals (95%) or mailed out (55%). Clinical or heart failure nurses were named as the health professionals handing out the materials in 86% of cases. The materials were explained to consumers in 97% of cases and were part of a larger education program in 28 cases (74%). Some organisations were using only one resource for consumers such as an information booklet on heart failure whilst others provided a combination of resources, such as an information booklet, a self-management diary and an action plan or fridge magnet. In some hospitals, delivery of consumer information resources was embedded as part of a global management plan. In one hospital, patients received information from a range of health professionals including occupational therapist, dietitian and cardiac rehabilitation practitioner. A hospital had produced a form for health professionals to record medical data but also to indicate whether the patient had received verbal education, watched an information video, understood how to manage and monitor symptoms and was compliant with prescribed medications. It also documented pharmacy and nutritional assessment notes, together with a list of questions to ask patients.

**Table 2:** Dissemination of consumer resources as reported in the 40 questionnaires

	Frequency* (%)
***Extent of distribution of resources***	
Locally	14 (37)
Regionally	10 (26)
State	6 (16)
Nationally	6 (16)
Other	2 (5)
***Settings where resources were distributed***	
Hospital department	30 (79)
Outpatient clinic	26 (68)
Rehabilitation centre	16 (42)
General practitioner surgery	7 (18)
Community pharmacy	0 (0)
Health information centre	2 (5)
Support group	6 (16)
Patient organisation	4 (10)
Patient home	9 (23)
***Mechanisms of distribution of resources***	
Handed out directly	36 (95)
Mailed out	21 (55)
Available in health information centres	4 (11)
Published in an organisation newsletter	4 (11)
Available on a web-site	7 (18)
Other	2 (5)
***Strategies used to inform consumers***	
Informed directly	37 (97)
Posters	4 (11)
Publicity through consumer groups	10 (26)
Publicity using media	9 (24)
Personal mailing	6 (16)
Website	6 (16)
Other	5 (13)
***Strategies used to inform health professionals***	
Local meetings	28 (76)
Institutional newsletters	14 (38)
Publicity through professional organisations	15 (41)
Publicity through media	9 (24)
Personal mailing	12 (32)
Website	8 (22)
Other	12 (32)

*Multiple responses could be given at each question, therefore percentages do not add up to 100%

Nearly all of the consumer materials were provided to consumers free of charge (95% of the cases). In 97% of cases, consumers were alerted to the availability of the resource by health professionals. Publicity through consumer groups and the media was rarely used. Health professionals were most commonly informed of the availability of consumer material through local meetings (76%). The majority of respondents (81%) indicated that they would be willing to share the consumer materials that they had produced. Approximately 75% of respondents used consumer information materials produced by another organisation/institution.

The questionnaire asked for suggestions to improve the dissemination of high quality consumer materials. Some respondents reported their concerns about the lack of sustainability when projects end, lack of standardisation and evidence-base, dependency on drug companies, duplication of efforts and lack of access to heart failure consumer resources by patients admitted to general medical wards. The most frequent suggestion for improving the dissemination of consumer resources was to develop a nationally coordinated approach and to set up a clearinghouse or website, easily accessible to both health professionals and consumers, which would identify high quality consumer materials and/or provide free access to them. Several respondents asked for increased availability of consumer materials through support groups, consumer advocates, cardiac rehabilitation programs, GP surgeries and Divisions. Several respondents mentioned the need to develop language specific materials and to develop audiovisuals for consumers from culturally and linguistically diverse backgrounds. Several respondents insisted that the distribution of consumer materials should be done by health professionals associated with health education to have an impact.

### Quality assessment of information booklets

A total of 21 information booklets were identified in the survey. Copies of 19 of these booklets were able to be obtained and assessed for their quality (Table 3).

**Table 3:** Information booklets on heart failure

Title	Organisation	Location	Description
Living with Heart Failure: information for you, your family and carers	Brisbane Cardiac Consortium	Queensland	A 45-page A4 booklet with information about heart physiology, epidemiology, causes, symptoms, diagnosis, treatment of heart failure, a checklist and when to seek medical help.
Let's Talk About Heart Failure	National Heart Foundation of Australia	National	A 16-page, colour A4 booklet covering symptoms, medications, lifestyle issues, physical activity and cardiac rehabilitation.
Your Guide to Heart Failure	Heart Failure Centre, Alfred Hospital	Victoria	A 24-page colour A4 booklet covering symptoms, causes, diagnostic procedures, lifestyle changes, medications and a glossary. Has been translated into 4 languages.
Your Guide to Heart Failure	Westbay Alliance	Victoria	A 26-page A4 booklet covering symptoms, causes, diagnostic procedures, lifestyle changes, medications and a glossary. Has been translated into 8 languages.
Living with Congestive Heart Failure	The Queen Elizabeth Hospital, University of Adelaide, North Western Adelaide Health Service	South Australia	A 31-page A4 booklet with information about heart physiology, causes, symptoms, diagnosis of heart failure, lifestyle issues and medications.
Heart Failure Management Plan	The Queen Elizabeth Hospital	South Australia	A 13-page A4 booklet with instructions for managing fluid intake and home exercise program.
Life Guide	Sir Charles Gairdner Hospital	Western Australia	A modular education resource that can be used for several cardiac conditions (including heart failure) by changing inserts in a folder. Divided into 6 sections: introduction, about the condition, health plan, medications, risk factors and community resources.
Heart Failure: patient information booklet	Advanced Heart Failure Service, Royal Perth Hospital	Western Australia	A 41-page A4 glossy booklet covering heart physiology, causes, diagnosis, classification, management, complications, emotional adjustments, medications, lifestyle and exercise, research, community support and a glossary.
Dilatrend Patient Support Service – Guide Book	Roche	National	A 60-page A5 booklet, includes 4 chapters on treating heart failure, living with heart failure, importance of healthy eating and importance of physical activity, also includes a glossary and useful contacts.
Getting to Know Your Medications	Prince of Wales Hospital	New South Wales	Presently a draft document, provides general information about medications and specific information on the medications actually taken.
A Patient Guide to Understanding and Managing Congestive Heart Failure	Mackay Base Hospital	Queensland	Pamphlet (photocopied) covering what is heart failure, treatment, diagnosis, signs and symptoms and management.
HEART Help Program	Bankstown Lidcombe Hospital	New South Wales	A series of information leaflets including treating congestive heart failure, a weight chart, fluid restriction, salt intake and eating right with warfarin (for patients taking warfarin).
Heart Failure: what you should know	Blacktown Hospital/Western Sydney Area Health Service	New South Wales	A4 double sided (photocopy) with information on causes and symptoms of heart failure and advice on "helping yourself'.
Heart Failure: what is it? (+ newsletter)	St George Hospital	New South Wales	An 8-page A5 booklet with information on what is heart failure, symptoms, self-management, and an action plan. A newsletter is also provided.
What can you do to help yourself?	Shoalhaven Heart Failure Service/Illawarra Area Health	New South Wales	Information leaflet giving advice on weight recording, healthy eating, lifestyle, exercise, medications, support groups.
Managing Congestive Heart Failure	Northern Rivers Area Health Service	New South Wales	16-page photocopied information booklet including what is CHF, diet, medications, exercise.
Heart Failure Information Package	Concord Repatriation Hospital/Central Sydney Area Health Service	New South Wales	6-page information booklet which includes causes, symptoms, non-drug management, medications and an action plan.
Your Lifestyle and Heart Failure & Medications and Heart Failure	Epworth Hospital	Victoria	4-page booklet covering diet, fluid restriction, alcohol, weight activity, exercise and smoking 2-page leaflet on medications
Cardiomyopathy information	Royal Adelaide Hospital	South Australia	A4 double-sided leaflet covering cardiomyopathy, symptoms, causes, treatment and what can be done.

Overall, the presentation and language of most of the information booklets/leaflets were judged to be appropriate for the intended consumer group (90% of the booklets) (Table 4), with legible print (95%) and clear headings (79%). The language and tone were found to be non-judgmental and likely to be understood. The majority was judged to avoid the use of global imperatives (95%). Only one booklet used a somewhat more dictating tone, discussing the "responsibilities" of the patient. There were important differences, however, in the overall presentation and design quality. Some were very attractive, making use of colour and photos, while others were poor quality black and white photocopies.

**Table 4:** Quality assessment of information booklets

**Criteria**	**Positive assessment %**
***Presentation***	
Is the print legible?	95
Is it appropriate for the intended consumer group?	90
Is the information presented in sections?	95
Do the sections have clear headings?	79
Is there suitable spacing between the individual sentences?	90
Is there suitable spacing between sections?	84
Do the diagrams included provide useful information?	78
Are the diagrams labelled?	33
Are the diagrams of an adequate size?	89
***Language***	
Is the language and tone used non-judgmental?	100
Is the language used likely to be understood by the consumers who use it?	100
Is the medical terminology, abbreviations, and jargon explained?	100
Is it written in the second person (for example, 'you' instead of 'the patient')?	100
Is the terminology used consistent? (that is, are the same words used to describe the same ideas, procedures or terms?)	100
Does the product avoid the use of global imperatives? (for example, will, should, must)	95
***Content***	
Is there evidence that consumers were involved in the production of the written consumer health information?	5
Are the aims or objectives of the product clearly stated?	32
Is the intended audience clearly stated in the product?	32
Does the product meet the specified aims?	83
Is information presented in a sequence that is useful to consumers, that is, is the most useful information presented first? (This may not necessarily be a logical sequence)	44
Is the information included in the product current?	79
Is the evidence provided in the product referenced?	0
Is the information presented in a balanced and non-biased way?	79
Are there any omissions that the consumer needs to be aware of?	74
Does the product provide information about areas of uncertainties in knowledge?	0
Has information about further sources of support and help been included?	32
Is the publisher included on the product?	95
Is the date of publication included?	53
Does the product contain the name or names of the author/s?	37
Are the credentials of the authors included?	21
Does the product encourage and support shared decision making or assist consumers to ask questions about their own treatment?	0
***Treatment options and outcomes***	
Are all the treatment options included?	33
Is there a description of all the treatment options?	28
Is there an indication of the quality and level of evidence to support these options?	6
Are treatment outcomes provided, including information about the risks and benefits?	6
Are the treatment outcomes quantified?	6
Is there a comparative analysis of the treatment choices?	0
Is there a balanced and unbiased description of the treatment options and outcomes?	6
	
Is there mention of what might happen if the no treatment option is selected?	6
Is information about the gaps and uncertainties in treatment provided?	0

Most booklets covered the same range of topics, including the physiology of the heart, pathophysiology of the disease, drug treatment, lifestyle issues and self-management advice. The extent of information provided on each topic varied with some booklets only providing a brief mention of a topic, while others covered the same topic in greater depth. One leaflet presented information on cardiomyopathy only. One booklet was about medications used in heart failure. Two leaflets were focused on a self-management plan, without detailed information on other topics. Information provided on medicines for heart failure was found to vary widely. In some cases this information was provided as a table with a list of side effects, whereas in other resources detailed information was provided, including general information on medicines and specific information on each therapeutic class. Variations were also noted in the advice given for exercise training and the emphasis placed on some aspects of exercise between different resources. Most information booklets told patients to call their doctor if they observe a weight increase. Very few gave instructions for self-management of diuretics.

Some aspects of the content that were judged generally to be done well were the provision of information in a balanced and non-biased way (79%) (Table 4). Some aspects that were judged to be less well done were the description of the aims of the resource (32%) and of the intended audience (32%). There was no evidence that consumers had been involved in the production of the booklet except in one case. No references were provided in any of the booklets. A majority of booklets (74%) had omissions that the consumer might need to be aware of. For example, some topics were only rarely or not covered: only one booklet gave epidemiological data on the disease albeit limited, no booklet gave information on prognosis of heart failure, two booklets gave information on emotional aspects, and none gave information on surgical treatment. No booklet provided information about areas of uncertainties in knowledge. Information about further sources of support and help was found in 32% of the booklets.

Only one booklet provided quantitative information about treatment outcomes and included the quality and level of evidence to support treatment options. None of the 19 booklets/leaflets provided a comparative analysis of treatment choices, and none gave information about the gaps and uncertainties in treatment provided.

An overall quality rating for information booklets was given according to a rating scale from 1 (high quality) to 5 (low quality): 8 booklets scored a rating of 2, 7 a rating of 3, 3 a rating 4, and 1 a rating of 5.

## Discussion

To our knowledge, this study is the only study which has assessed the availability of consumer information resources for patients with heart failure at the national level. Some studies have assessed the quality of information resources for other diseases [6,10,11] but none has looked at the dissemination of these resources.

Our survey identified 61 different consumer resources on heart failure, most of which were information booklets, self-management diaries or self-management action plans. This represents a significant number and variety of consumer resources developed in Australia in recent years. However, as this seems to have been encouraged by the implementation of short-term local disease management projects, the issues of sustainability and currency of consumer resources were raised by several respondents.

Dependency on drug company funding was also identified as a contentious area with the main consumer resources in 2 states partially or totally funded by drug companies, as well as the 3 videotapes identified. Materials produced with such funding may tend to be biased in favour of the company's products [12]. Reliance on drug companies' funding may obligate health professionals to reciprocate in some way such as in increasing the prescriptions of the company's products [13].

Our findings also showed that dissemination of consumer resources was limited in most states. More than half of the consumer resources had been developed in 2 states only. Regional hospitals seemed to have a more limited range of resources available to use with their patients than metropolitan hospitals. Consumer resources specifically developed for dissemination in general practice were limited to a self-management plan on a website and a brief, out-dated, print-out available in a prescribing software. Health services may have access to national resources, such as the National Heart Foundation booklet but may not have the structures to allow them to use resources as part of specific disease management programmes. In addition, some patients who have a diagnosis of heart failure may never come into contact with specialised services where they would receive such resources. In 2004–5, only a minority (8%) of Australian patients were managed in chronic heart failure management programs [14].

Strategies to improve the availability and dissemination of heart failure consumer resources across the whole range of health care settings as well as support groups and carers' organisations need to be explored. The most frequent suggestion made by respondents to both questionnaires was to develop a nationally coordinated approach and set up a database, clearinghouse or website easily accessible to both health professionals and consumers, which identifies high quality consumer resources and/or provides free access to them. In February 2004, in response to the findings of this study, the National Institute of Clinical Studies (NICS) launched an online directory to feature the four highest quality information booklets identified in this study. However, the directory was discontinued two years later when an evaluation showed a low level of awareness of the directory amongst health care professionals (< 10%) and a low level of usage of the directory amongst those who were aware of the directory [15]. Our study coincided with the national launch of the National Heart Foundation booklet. In 2008, this booklet was updated and tested among patients. It is likely that the availability of this booklet may have changed the patterns of production and use of consumer information on heart failure in Australia in the recent years. However, the dissemination of consumer information resources on heart failure, especially by general practitioners, remains a critical issue. A 2006 survey of 240 Australian GPs showed that 73.9% did not provide any written information on heart failure, 15.1% provided the Heart Foundation booklet and 10.9% provided other types of information, mainly the prescribing software heart failure leaflet [15]. The provision of information on the Internet is helpful but cannot be the only answer. Provision of consumer information needs to be integrated into medical care, and health services need to be designed to make consumer information a key commodity accessible to all patients as is the case for other health care commodities such as medicines and diagnostic tests [16].

The quality assessment of 19 information booklets showed that, overall, the presentation and language were appropriate and that information was provided in a balanced and non-biased way in most booklets. There were gaps in terms of topics covered (such as epidemiology of the disease, prognosis, emotional aspects), and types of information included, such as references, areas of uncertainties in knowledge, quantitative information about treatment outcomes and information on the quality and level of scientific evidence to support medical recommendations. However, it should be acknowledged that the balance between comprehensiveness of the information provided and the ability to be understood by patients with a wide range of literacy levels and information needs is difficult to achieve.

There was no explicit indication that consumers had been involved in the production of the booklets except in one case. Consumer involvement may in fact have been higher as respondents to the questionnaires reported that consumers had some type of input in two thirds of the consumer resources identified. The patient's expanding role in making decisions and the complexity of treatment options require that consumer resources be developed to facilitate the patient's informed choice and self-management behaviour [16]. While it is generally agreed that consumer resources should be based on a comprehensive evaluation of the research related to the specific illness [17], consumer resources cannot be a simple translation of clinical guidelines. Several studies have shown an ontological dissociation between clinical observations of heart failure symptomatology and patients' beliefs and interpretations of their experiences of the disease [18,19]. Granger suggested that more targeted clinical interventions should be developed by learning to think differently from both the patient and provider perspectives [18]. Patient education should aim to close the gap between patients' experiences of illness and the clinical interpretation of the disease while targeting barriers to learning such as misconceptions and functional and cognitive limitations [20]. It is therefore essential that consumers are actively engaged in the production of all consumer information materials.

## Limitations

While this study aimed to provide an accurate picture of the availability of consumer resources in Australia in contacting a wide range of health information providers including all hospitals in the major metropolitan centers, we did not monitor the proportion of patients with heart failure actually receiving information on heart failure. Moreover, many patients are managed mainly by their general practitioner and may not come near a specialty service, although the situation may change in the future with the funding of heart failure community services in states such as Queensland [2].

We used a standardized checklist to assess the quality of information booklets. However, there is always a level of subjectivity involved in rating criteria such as the completeness or the currency of the information provided. In the King's Fund seminal report on the assessment of the quality of patient information materials, patients and specialists were rating separately the quality of patient information materials on various health problems [10]. They did not always agree and in some cases the materials were ranked quite differently by the two groups. In our study, the quality assessment was undertaken by a drug information pharmacist with extensive skills in critical appraisal of the health literature. Patients, however, are the only ones who can assess how easy, or hard, it is for them to read and understand the information and how personally relevant, or useful, the information is.

Another limitation is that the assessment focused on the quality of the information booklets only, whereas several hospitals were using a set of consumer resources, rather than a single resource, such as an information booklet plus a self-management action plan and patient diary, as well as allied health leaflets on exercise training and medications. The quality of the information provided in those hospitals cannot be judged on the quality rating obtained for the information booklet alone.

## Conclusion

Consumer information is considered as an essential part of patient education. It provides support for informed choice and self-management behaviour and is a key component of all disease management programmes of patients with heart failure. The dissemination and the quality of consumer resources on heart failure in Australia still need to be enhanced to improve patient outcomes and answer the mounting need for better quality patient information.

## Competing interests

The authors declare that they have no competing interests.

## Authors' contributions

AIV and SJS designed the study, collected, analysed and interpreted data and drafted the manuscript. SMP had the idea of the study, participated in the design and the interpretation of the results, and drafted the manuscript. All authors read and approved the final manuscript.

## Pre-publication history

The pre-publication history for this paper can be accessed here:



## References

[B1] Krum H, Tonkin AM, Currie R, Djundjek R, Johnston CI (2001). Chronic heart failure in Australian general practice. The Cardiac Awareness Survey and Evaluation (CASE) Study. Med J Aust.

[B2] Stromberg A (2005). The crucial role of patient education in heart failure. Eur J Heart Fail.

[B3] Lainscak M, Keber I (2006). Patients' knowledge and beta blocker treatment improve prognosis of patients from a heart failure clinic. Eur J Heart Fail.

[B4] Jovicic A, Holroyd-Leduc JM, Straus SE (2006). Effects of self-management intervention on health outcomes of patients with heart failure: a systematic review of randomized controlled trials. BMC Cardiovasc Disord.

[B5] Coulter A, Entwistle V, Gilbert D (1999). Sharing decisions with patients: is the information good enough?. BMJ.

[B6] Currie K, Rajendran M, Spink J, Carter M, Anderson J (2001). Consumer health information. What the research is telling us. Aust Fam Physician.

[B7] Currie K, Spink J, Rajendran M (2000). Communicating with consumers series, Volume 1, Well-written health information guide.

[B8] Buetow SA, Coster GD (2001). Do general practice patients with heart failure understand its nature and seriousness, and want improved information?. Patient Educ Couns.

[B9] Ni HND, Burgess D, Wise K, Crispell K, Hershberger RE (1999). Factors influencing knowledge of and adherence to self-care among patients with heart failure. Arch Intern Med.

[B10] Coulter A (1999). Informing patients. An assessment of the quality of patient information materials. BMJ.

[B11] Rees CE, Ford JE, Sheard CE (2003). Patient information leaflets for prostate cancer: which leaflets should healthcare professionals recommend?. Patient Educ Couns.

[B12] Garlick W (2003). Should drug companies be allowed to talk directly to patients?. BMJ.

[B13] Dana JLG (2003). A social science perspective on gifts to physicians from industry. JAMA.

[B14] Clark RA, Driscoll A, Nottage J, McLennan S, Coombe DM, Bamford EJ, Wilkinson D, Stewart S (2007). Inequitable provision of optimal services for patients with chronic heart failure: a national geo-mapping study. Med J Aust.

[B15] Vitry A, Semple S (2006). Evaluation of the NICS online directory of heart failure patient resources.

[B16] Woolf SH, Chan EC, Harris R, Sheridan SL, Braddock CH, Kaplan RM, Krist A, O'Connor AM, Tunis S (2005). Promoting informed choice: transforming health care to dispense knowlege for decision making. Ann Intern Med.

[B17] Toman C, Harrison MB, Logan J (2001). Clinical practice guidelines: necessary but not sufficient for evidence-based patient education and counseling. Patient Educ Couns.

[B18] Granger BB, Moser D, Germino B, Harrell J, Ekman I (2006). Caring for patients with chronic heart failure: The trajectory model. Eur J Cardiovasc Nurs.

[B19] Field K, Ziebland S, McPherson A, Lehman R (2006). 'Can I come off the tablets now?' A qualitative analysis of heart failure patients' understanding of their medication. Fam Pract.

[B20] Riegel B, Vaughan Dickson V, Goldberg LR, Deatrick JA (2007). Factors associated with the development of expertise in heart failure self-care. Nurs Res.

